# Human T-Cell Lymphotropic Virus: A Model of NF-κB-Associated Tumorigenesis

**DOI:** 10.3390/v3060714

**Published:** 2011-06-10

**Authors:** Zhaoxia Qu, Gutian Xiao

**Affiliations:** 1 Cancer Institute, Medical Center, University of Pittsburgh, Pittsburgh, PA 15213, USA;; 2 Department of Microbiology and Molecular Genetics, School of Medicine, University of Pittsburgh, Pittsburgh, PA 15261, USA

**Keywords:** ATL, IKK, HTLV, immune escape, NF-κB, PDLIM2, Tax, transformation, tumorigenesis, virus-host interaction, WWOX

## Abstract

Human T-cell lymphotropic virus type 1 (HTLV-1) is the etiological agent of adult T-cell leukemia/lymphoma (ATL), whereas the highly related HTLV-2 is not associated with ATL or other cancers. In addition to ATL leukemogenesis, studies of the HTLV viruses also provide an exceptional model for understanding basic pathogenic mechanisms of virus-host interactions and human oncogenesis. Accumulating evidence suggests that the viral regulatory protein Tax and host inflammatory transcription factor NF-κB are largely responsible for the different pathogenic potentials of HTLV-1 and HTLV-2. Here, we discuss the molecular mechanisms of HTLV-1 oncogenic pathogenesis with a focus on the interplay between the Tax oncoprotein and NF-κB pro-oncogenic signaling. We also outline some of the most intriguing and outstanding questions in the fields of HTLV and NF-κB. Answers to those questions will greatly advance our understanding of ATL leukemogenesis and other NF-κB-associated tumorigenesis and will help us design personalized cancer therapies.

## 1. Introduction

**Figure 1.**
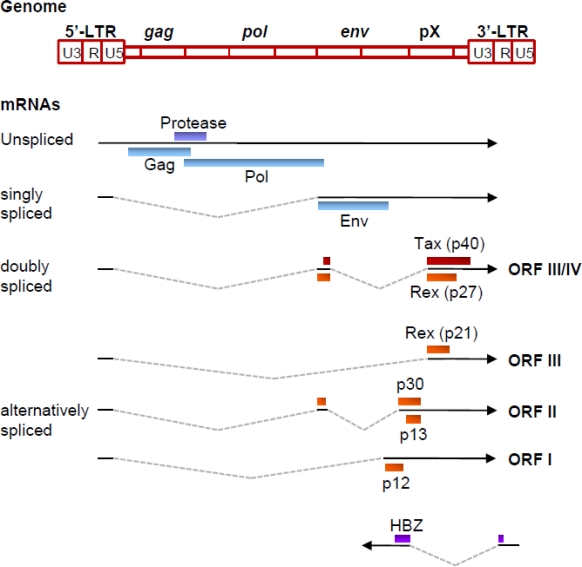
The Human T-cell lymphotropic virus (HTLV) proviral genome. Gag, Pol, and Env are viral structural proteins, others are viral regulatory/accessory proteins. Except the *hbz* gene, which is encoded by the minus strand of the HTLV proviral genome from 3′-LTR, all other genes are encoded by the plus strand under the direction of the 5′-LTR. Of note, the 5′-LTR is frequently deleted or methylated as disease progresses. In addition, the *tax* gene often undergoes nonsense or missense mutations during the late stages of ATL leukemogenesis. Although the Tax protein and the *hbz* gene induce tumors in transgenic mice and p12 shows weak oncogenic activity *in vitro* [17–23,245,260], none of the viral proteins/genes except Tax are required for HTLV-1-mediated tumorigenesis [16,261–263]. ORF: open reading frame.

Human T-cell leukemia virus type 1 (HTLV-1) and type 2 (HTLV-2) are closely related human retroviruses that were originally discovered in the early 1980s [1]. They have a similar genome structure with approximately 70% nucleotide homology (Figure 1). They also share a common mechanism of replication and transmission. But the pathogenic potentials and clinical manifestations of these two highly related viruses differ significantly [2]. HTLV-1 is the etiological agent of adult T-cell leukemia/lymphoma (ATL), an aggressive and lethal malignancy of CD4^+^ T lymphocytes, as well as a variety of autoimmune and inflammatory diseases including the neurodegenerative disorder tropical spastic paraparesis/HTLV-1-associated myelopathy (TSP/HAM). However, no significant association of HTLV-2 with human malignancies has been demonstrated. Unfortunately, there is still no cure for HTLV-1-associated malignancies and no means of assessing the risk of disease or prognosis in infected people [3–5]. In addition to the direct clinical problems caused by HTLV-1 infection, studies of HTLV-1 particularly in comparison with HTLV-2, provide important models for understanding basic pathogenic mechanisms of host-virus interaction, human oncogenesis, and inflammatory and autoimmune disorders.

**Figure 2.**
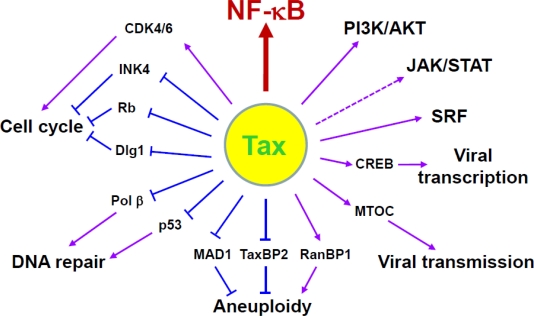
Cellular targets of the Tax viral oncoprotein. NF-κB, PI3K/AKT and SRF are well-known for their roles in various cellular functions, particularly cell survival and cell proliferation. The JAK/STAT signaling pathway is activated indirectly through Tax-dependent cytokine induction, while all other signaling molecules/signaling pathways are directly regulated by Tax. SRF: serum response factor.

Unlike animal oncoretroviruses, HTLV-1 does not carry a host-derived oncogene or activate a cellular oncogene through proviral integration [6]. Instead, HTLV-1 encodes a regulatory protein Tax that serves as the primary oncogenic mediator [7–9]. Tax not only transforms rodent fibroblasts but also immortalizes human primary T cells *in vitro* [10–13]. Compared to cells transformed by many cellular oncogenes, Tax-transformed cells have an apparently higher resistance to the induction of apoptosis [14]. In addition, Tax-transformed lymphoid cells and fibroblasts induce tumors when introduced into immunodeficient mice (nude mice or SCID mice) [10,13,15]. More importantly, the HTLV-1 genome without Tax loses its original transforming ability [16], whereas Tax transgenic mice develop various tumors depending on the type of the promoters used to drive Tax expression [17–23]. A more recent study shows that Tax-transduced human hematopoietic stem cells, a preferential HTLV-1 reservoir *in vivo*, acquire the ability to develop CD4^+^ T-cell lymphomas in SCID mice [24]. Of note, Tax-immortalized lymphocytes *in vitro* and Tax-mediated T-cell lymphoma in animals closely resemble the phenotype of HTLV-1-transformed T-cells and HTLV-1-induced ATL, respectively [23–25]. Tax is a pleiotropic protein that exploits various cellular machinery and signaling pathways to mediate cellular transformation as well as viral replication (Figure 2). Among those host machineries, NF-κB signaling plays a pivotal role in Tax-mediated transformation and ATL leukemogenesis.

## 2. NF-κB Signaling Pathways

### 2.1. The NF-κB Family

**Figure 3.**
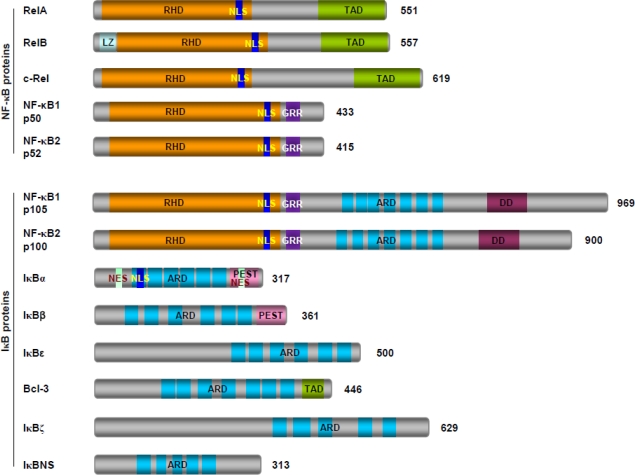
Schematic representation of members of NF-κB and IκB families. ARD: ankyrin repeat domain; DD: death domain; GRR: glycine-rich region; LZ: leucine zipper; NES: nuclear export sequence; NLS: nuclear localization sequences; PEST: PEST containing sequence; RHD: Rel homology domain; TAD: transactivating domain. The NF-κB family can be divided into two subfamilies. One subfamily consists of three members: RelA, RelB and c-Rel; and the other one contains two members: NF-κB1/p50 and NF-κB2/p52. Typical NF-κB dimers are usually composed of one member from each subfamily, such as RelA/p50 and RelB/p52, although all NF-κB members may form various homo- or hetero-dimers. Of note, the p50 or p52 homodimers mainly function as transcription repressors due to lack of a TAD. The IκB family can be classified into three subfamilies: the typical IκB proteins (IκBα and IκBɛ), the precursor proteins (p100 and p105) and the atypical IκB proteins (BCL-3, IκBβ, IκBζ and IκBNS). The typical subfamily simply functions as NF-κB inhibitors. In addition to being NF-κB inhibitors, the precursor subfamily is also required to generate the NF-κB members p50 and p52. The atypical subfamily may function as a co-activator or co-repressor of NF-κB depending on the situation. When binding to Bcl-3, the p50 or p52 homodimers can also induce gene transcription.

NF-κB, nuclear factor-κB, is a family of transcription factors that plays a central role in the regulation of diverse biological processes, including immune responses, development, cell proliferation and survival [26]. Deregulated NF-κB has been linked to a variety of human diseases, particularly cancers [27]. The NF-κB family consists of five closely related DNA binding proteins: RelA (p65), RelB, c-Rel, NF-κB1/p50 and NF-κB2/p52, which function as various homodimers and heterodimers to regulate transcription of genes containing κB motifs in their promoters [26]. NF-κB members share a highly conserved 300-amino acid-long N-terminal Rel homology domain (RHD), which is responsible for their dimerization, nuclear translocation, DNA binding and also interaction with the inhibitors of NF-κB (IκBs) (Figure 3). However, NF-κB family members exhibit major differences in their C-terminal sequences as well as in their modes of synthesis. RelA, RelB and c-Rel have transactivating domains (TAD) at their C-termini and are synthesized directly as mature forms, whereas p50 and p52 lack a TAD and are generated from large precursor proteins, p105 and p100, respectively. Interestingly, p105 and p100 contain IκB-like sequences in their C-terminal portions and function as NF-κB inhibitors [28,29]. Processing of p105 and p100 (selective degradation of their C-terminal IκB-like sequences) thus has two functions: to disrupt their IκB-like function and to generate mature NF-κB subunits. Since p105 is constitutively processed to p50 and is usually completely degraded upon NF-κB stimulation [30,31], it can be simply considered as a “typical” IκB. On the other hand, p100 processing is tightly controlled and its induction is highly signal-dependent [32,33].

### 2.2. Pathways Leading to NF-κB Activation

**Figure 4.**
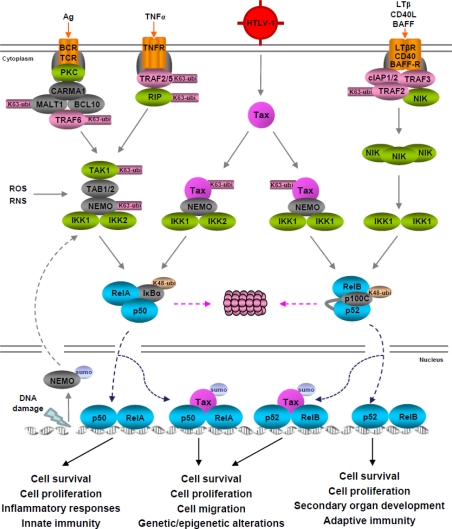
NF-κB signaling pathways. Although the canonical and non-canonical signaling pathways primarily activate the RelA/p50 and RelB/p52 dimers, respectively, all NF-κB members may be activated by either pathway or both. In fact, the RelA/p50 dimers may be sequestered in the cytoplasm by p100 and can be activated through p100 processing. On the other hand, NF-κB dimers containing p52 may be sequestered in the cytoplasm by IκBα and can be activated through IκBα degradation. Furthermore, activation of the canonical NF-κB signaling pathway can be induced through inducible degradation of IκBβ, IκBɛ and p105, a process similar to the inducible IκBα degradation, although their degradation dynamics can be different.

In unstimulated cells, NF-κB dimers are usually sequestered in the cytoplasm by IκBs such as IκBα and p100. NF-κB nuclear translocation and subsequent transcription activation require degradation of IκBs or processing of p100 to generate p52, which represent two major mechanisms of NF-κB activation [26]. Due to the fundamental difference between inducible IκB degradation and p100 processing in their signal transduction and biological outcomes, the two mechanisms leading to NF-κB activation are termed as the canonical (classical) and non-canonical (non-classical) NF-κB pathways, respectively (Figure 4).

#### Canonical NF-κB pathway:

The canonical pathway can be rapidly activated by a plethora of stimuli from either outside or inside cells, such as extracellular antigens and inflammation cytokines (e.g., tumor necrosis factor, TNF, a prototypic NF-κB stimulus), cytoplasmic oxidative stress and nuclear DNA damage [34]. These stimuli induce assembly of a multimolecular complex that includes the RING-finger E3 ubiquitin ligase TNF receptor associated factor 6 (TRAF6) or other TRAF proteins, leading to K63-linked auto-polyubiquitination of TRAF6 [35,36]. The ubiquitinated TRAF6 recruits and catalyzes K63-linked ubiquitination of the transforming growth factor-β-activated kinase 1 (TAK1) and the IκB kinase (IKK) complex (the IKK complex consists of two catalytic components, IKK1 (IKKα) and IKK2 (IKKβ), and a regulatory component, NEMO (NF-κB essential modulator, IKKγ)), so that TAK1 can phosphorylate and activate IKK [37]. Once activated, IKK phosphorylates specific serines within IκBs (e.g., IκBα, S32 and S36; IκBβ, S19 and S23; p105, S927 and S932), triggering their K48-linked ubiquitination by the E3 ubiquitin ligase β-transducin repeat-containing protein (β-TrCP) and subsequent degradation by the 26S proteasome [26,27]. NF-κB released from IκBs then translocates into the nucleus to regulate expression of a wide range of genes, particularly those involved in cell proliferation, survival, adhesion and migration [34]. In addition to IκB degradation, many other regulatory mechanisms are also important for canonical NF-κB activation, such as phosphorylation, prolyl isomerization and acetylation of RelA [26,27]. These post-translational modifications prevent RelA from binding to IκBα, facilitate RelA to recruit the transcriptional coactivators CBP/p300, and/or increase the DNA binding activity and protein stability of RelA [38–41].

#### Non-canonical NF-κB pathway:

In contrast to the canonical pathway, the noncanonical NF-κB pathway is induced only by a handful of stimuli including B-cell activating factor (BAFF), lymphotoxin β (LTβ), CD40 ligand (CD40L), TNF-like weak inducer of apoptosis (TWEAK), and receptor activator of NF-κB ligand (RANKL) [26]. In addition, activation of the noncanonical NF-κB pathway is slow and depends on protein synthesis of NF-κB-inducing kinase (NIK) [32,42]. Although its mRNA expression is relatively abundant, the level of NIK protein is normally very low because it is constitutively degraded via a TRAF3-dependent mechanism [42,43]. TRAF3 functions as a scaffold between NIK and TRAF2, which in turn recruits cellular inhibitors of apoptosis 1 and 2 (c-IAP1/2) into the NIK complex. Within the complex, c-IAP1 or c-IAP2 acts as the E3 ubiquitin ligase to mediate NIK polyubiquitination and proteolysis, thereby keeping its abundance below the threshold required for its function [44]. In response to noncanonical NF-κB stimuli, either TRAF2 and TRAF3 or c-IAP1 and c-IAP2 are degraded by the proteasome, resulting in stabilization and accumulation of the newly synthesized NIK, thereby allowing NIK proteins to form oligomers and cross-phosphorylate each other for their activation [42,43,45–51]. Self-activated NIK in turn activates the IKK complex and specifically recruits IKK1 into the p100 complex to phosphorylate p100, leading to p100 ubiquitination by the β-TrCP E3 ubiquitin ligase and processing by the proteasome to generate p52 [32,52–54]. The processed p52 product, together with its NF-κB binding partner, translocates into the nucleus to induce or repress gene expression. Moreover, NIK-activated IKK may also induce IκBα degradation to activate the canonical NF-κB pathway [55].

### 2.3. Termination of NF-κB Activation

Activation of the NF-κB pathways is tightly regulated and rapidly curtailed following the initial activating stimulus. Transient activation of NF-κB is physiologically important because persistent activation can result in deleterious or even fatal conditions, such as acute inflammation, septic shock or at a cellular level, inappropriate cell growth and survival leading to cancer [26]. It is therefore not surprising that feedback inhibition mechanisms to terminate NF-κB activation occur at almost all steps in the leading to activation.

Consistent with the central role of IKK in the activation of both canonical and non-canonical NF-κB pathways, several mechanisms are employed to inactivate IKK. Once activated, IKK phosphorylates itself and its upstream activators, such as RIP in the canonical NF-κB pathway and NIK in the non-canonical NF-κB pathway, in addition to the IκB proteins. The autophosphorylation of the IKK catalytic components at their multiple C-terminal serines is supposed to cause conformational alteration of IKK and phosphatase recruitment, resulting in dephosphorylation of the IKK activation loops and IKK inactivation [56]. Phosphorylation of RIP and NIK, similar to IκB phosphorylation, leads to K48-linked ubiquitination and degradation of these IKK activators [57,58]. The ubiquitination of RIP is mediated by A20 (TNFAIP3, TNFα-induced protein 3), a known target of NF-κB activation [59], providing a distinct feedback inhibition mechanism. In addition to functioning as an E3 ubiquitin ligase for RIP K48-linked ubiquitination and degradation, A20 exerts at least two additional functions to terminate NF-κB activation. First it can function as a deubiquitinase (DUB) to remove K63-linked ubiquitin chains from multiple NF-κB signaling molecules such as TRAF2/6, RIP, MALT1 and NEMO. Alternatively, it can block continuous K63-linked ubiquitination of these key NF-κB regulators by disrupting the interaction between the K63 ubiquitin ligases TRAF2/6 and their E2 ubiquitin conjugating enzymes Ubc13 and UbcH5c [58,60–63]. As stated above and shown in Figure 4, K63-linked ubiquitination of NF-κB signaling molecules is critical for the assembly of signaling complexes and subsequent activation of IKK/NF-κB. Interestingly, A20 is also a target of IKK activation for phosphorylation. In this case, IKK-mediated phosphorylation increases the K63-specific DUB activity of A20, suggesting another feedback inhibition mechanism of IKK/NF-κB activation [64]. Besides A20, another deubiquitinase termed cylindromatosis (CYLD) also plays an important role in the termination of IKK/NF-κB activation [65]. Like A20, CYLD is a target gene of NF-κB activation and can remove K63-linked ubiquitin chains from multiple activated IKK/NF-κB signaling molecules, including TRAF2/6, RIP, TAK1, NEMO and Bcl-3 [66–68].

Given the role of RelA posttranslational modifications in its transcriptional activity, several mechanisms have been reported to reverse these modifications for NF-κB termination. For example, phosphorylation and acetylation of RelA are reversibly regulated by different phosphatases and histone deacetylases (HDACs) [38,39,41,69–72]. Moreover, RelA phosphorylation induced by pro-inflammatory cytokines is blocked by a protein called SINK and the DNA binding activity of RelA can be prevented by the basic helix-loop-helix (bHLH) transcription factor Twist or RelA-associated inhibitor (RAI) through their associations with RelA in the nucleus [73–75]. Interestingly, SINK and Twist are known target genes of NF-κB activation [73,74], suggesting that feedback inhibition is a common mechanism for NF-κB termination at different levels.

The best known and most critical feedback inhibition mechanism is to replenish the pool of IκB proteins via NF-κB activation. Similar to other NF-κB repressors, all IκB family members except IκBβ are direct targets of NF-κB. In particular, newly synthesized IκBα enters the nucleus to bind to and transport NF-κB dimers back to the cytoplasm to reconstitute the status quo ante [76].

Recent studies indicate that this feedback inhibition mechanism is neither sufficient nor necessary to turnoff NF-κB activation, at least in certain situations [77]. Instead, ubiquitination-mediated degradation of nuclear NF-κB provides a more rapid and essential mechanism for NF-κB termination. In this context, PDZ-LIM domain-containing protein 2, PDLIM2, a ubiquitously expressed nuclear protein with a strong cytoplasmic-nuclear shuttling activity, is particularly important. PDLIM2 terminates NF-κB activation using two distinct but related mechanisms: it not only functions as an E3 ubiquitin ligase to promote nuclear RelA ubiquitination but also shuttles RelA to the nuclear matrix for the proteasome-mediated degradation [78,79]. Importantly, PDLIM2 knockout mice are more sensitive to septic shock due to enhanced p65 activation and subsequently augmented production of inflammatory cytokines [78].

## 3. HTLV-1 Deregulation of NF-κB

Although tightly controlled in normal cells including T cells, NF-κB is constitutively activated in both transformed and untransformed HTLV-1-infected cells [80]. Given the association of NF-κB activation with tumorigenesis and the oncogenic ability of Tax [27], much effort has been devoted to elucidating the mechanism by which Tax persistently activates NF-κB. In fact, Tax is the first pathogenic agent shown to activate NF-κB, and the studies on Tax have greatly advanced our understanding of both physiological and pathogenic activations of NF-κB.

### 3.1. Tax-Mediated NF-κB Activation

#### Activation of the canonical NF-κB pathway by Tax:

The initial clue suggesting a role of the Tax oncoprotein in NF-κB activation came from the findings that Tax is able to activate the κB element in the promoter of the interleukin 2 (IL2) receptor alpha (IL-2Rα) gene and in the long terminal repeat (LTR) of the human immunodeficiency virus type 1 (HIV-1) [81–84]. Since then, our knowledge of Tax activation of NF-κB has increased significantly. We now know that Tax intervenes at multiple levels to activate NF-κB. In the cytoplasm, Tax directly binds to the IKK regulatory component NEMO, via the leucine-repeat motif of Tax and two homologous leucine zipper domains within NEMO, and recruits the IKK complex to the perinuclear compartment where IKK is phosphorylated and activated [85–88]. The activated IKK in turn phosphorylates IκBs (by IKK2) and also RelA (by IKK1), resulting in ubiquitination and proteasomal degradation of IκBs and subsequent nuclear translocation of NF-κB including the phosphorylated RelA [89]. In the nucleus, Tax recruits RelA as well as other cellular transcriptional components into interchromatin granules to form discrete transcriptional hot spots termed ‘Tax nuclear bodies’ for full NF-κB transcriptional activation [90,91].

Currently, the detailed mechanism of how the Tax-IKK interaction activates IKK remains largely unknown. Tax does not have kinase activity and cannot directly phosphorylate IKK for its activation. Given the dimerization ability of Tax [92,93], one possibility is that through self-dimerization, Tax brings different IKK complexes together so that they can cross-phosphorylate and activate each other. In support of the hypothesis, fusion of Tax, but not its M22 mutant that is defective in self-dimerization, to IKK1 or IKK2 is sufficient for their catalytic activation [94]. Tax may also act as an adaptor protein to recruit the IKK complex and its upstream kinase to the perinuclear compartment to form a new complex for IKK phosphorylation and activation. In this regard, the mitogen-activated protein kinase kinase kinases (MAP3Ks), MEKK1, NIK, Tpl2, and TAK1, have been shown to interact with Tax and enhance Tax-mediated IKK activation when over-expressed [95–98]. However, other studies suggest that none of these kinases is required for Tax-mediated IKK activation [99–101]. Instead, Tax may activate these MAP3Ks for activation of signaling pathways other than IKK/NF-κB. Another debated issue is the subcellular locations for Tax-mediated IKK activation. Some suggest it is the centrosome [102], while others imply endoplasmic reticulum or Golgi-associated structures [103–106].

Interestingly, the critical cytoplasmic and nuclear steps of NF-κB activation seem to involve two distinct posttranslational modifications of Tax protein, K63-linked ubiquitination and sumoylation, respectively [107,108]. While the K63-linked ubiquitination of Tax is mediated by the E2 ubiquitin conjugating enzyme Ubc13 and E3 ubiquitin ligase TRAF2, 5 or 6 [95,109], the E3 sumo ligase for Tax sumoylation has not yet been identified. Both ubiquitination and sumoylation of Tax involve the same C-terminal lysines, suggesting exclusive mechanisms for the two modifications [102,107,108]. Currently, it remains unclear whether the same Tax proteins undergo two different modifications for cytoplasmic-nuclear shuttling to exert their cytoplasmic and nuclear functions in the IKK/NF-κB activation, or whether different Tax proteins are involved in the different modifications and functions. A recent study suggests that the same Tax molecule alternatively undergoes ubiquitination at the centrosome or sumoylation at Tax nuclear bodies, and shuttles between these cytoplasmic and nuclear compartments [110]. Interestingly, the same study suggests that the ubiquitination and sumoylation of Tax also controls the shuttling of NEMO proteins among the centrosome and different Tax nuclear bodies and facilitates NEMO sumoylation in Tax nuclear bodies when over-expressed. Nuclear shuttling and sumoylation of NEMO are key steps for nuclear initiated IKK/NF-κB activation such as by DNA damage, an event particularly important for cancer biology and cancer treatment [111]. NEMO sumoylation induced by DNA damage triggers NEMO phosphorylation and monoubiquitination, which in turn leads to the relocation of NEMO back to the cytoplasm where the IKK-activating kinase TAK1 is recruited to phosphorylate IKK for its catalytic activation [111]. Thus, it is interesting to examine whether nuclear sumoylation of NEMO happens under HTLV-1 pathogenic conditions and whether Tax-induced NEMO sumoylation is also involved in the induction of NEMO ubiquitination, TAK1 recruitment and IKK catalytic activation. This idea may be challenged by previous studies showing that fusion of the NEMO N-terminus, which is responsible for the NEMO/IKK1/2 interaction but lacks the sumoylation or ubiquitination sites [112], to Tax is sufficient to activate IKK/NF-κB in NEMO deficient cells [94]. In light of this, some studies suggest that Tax-mediated IKK activation is independent of NEMO K63-linked ubiquitination and IKK upstream kinases including TAK1 [99,100]. Furthermore, Tax-induced NEMO sumoylation actually reduces the ubiquitination of NEMO proteins [110]. Those studies strongly argue against the role of NEMO nuclear sumoylation in Tax-mediated IKK activation. Alternatively, Tax-induced NEMO sumoylation may prevent the nuclear function of NEMO and therefore contribute to the transcriptional activation of NF-κB. In this regard, it has been reported that NEMO can translocate into the nucleus to repress NF-κB-mediated gene transcription by competing with RelA for the transcriptional co-activator CBP [113].

Besides the ubiquitin and sumo modifications, Tax also undergoes phosphorylation and acetylation [114–116]. Although the kinase(s) responsible for Tax phosphorylation remain to be identified and the involved phosphorylation sites are still controversial [114,116], Tax phosphorylation seems to be important for NF-κB activation, possibly by contributing to Tax nuclear translocation, and subsequent sumoylation and acetylation in the Tax nuclear bodies [115]. Furthermore, the phosphorylation of Tax may be involved in Tax binding to the prolyl isomerase Pin1 and subsequent Tax protein stabilization [117,118]. Previous studies have shown that Pin1 directly interacts with and stabilizes phosphorylated RelA and c-Rel, thereby increasing NF-κB activity and promoting oncogenesis [40,119]. Thus, it is of interest to examine whether Tax recruitment of Pin1 stabilizes RelA and other NF-κB members, besides Tax itself.

#### Activation of the noncanonical NF-κB pathway by Tax:

In addition to activation of the canonical NF-κB pathway, Tax induces the processing of p100 to yield p52 for the activation of the noncanonical NF-κB pathway [26]. The induction of p100 processing is a hallmark of NF-κB activation by HTLV-1 infection because activation of this alternative pathway usually occurs in B cells and lymphoid stromal cells but not in either resting or activated normal T cells [120]. In contrast to the physiological processing of p100, which requires the NIK kinase but is independent of NEMO, Tax activation of the noncanonical NF-κB pathway requires NEMO but is independent of NIK [120]. NEMO is required in this pathogenic process is because it plays an adaptor role in the assembly of the Tax/IKK complexes [120], a step also required to activate the canonical NF-κB pathway [121,122]. However, unlike the canonical Tax/NEMO/IKK complex, which contains both IKK1 and IKK2, the noncanonical Tax/NEMO/IKK complex only contains IKK1, but not IKK2 [120]. Like the NIK kinase, the physiological stimulator of p100 processing, Tax not only activates IKK1 but also recruits IKK1 (indirectly via NEMO) into the p100 complex. Within the p100 complex, IKK1 phosphorylates p100, leading to p100 ubiquitination and processing by the β-TrCP ubiquitin ligase and the proteasome, respectively [123].

### 3.2. Tax-Independent NF-κB Activation

Obviously, Tax-mediated IKK activation is a major mechanism contributing to the high NF-κB activation in HTLV-1-infected cells. However, Tax expression is lost in about 60% of all ATLs during the late stages of leukemogenesis because of hypermethylation, deletion of the proviral 5′ LTR, or nonsense or missense mutations of the *tax* gene [8,124–129]. Notably, both canonical and noncanonical NF-κB pathways are still strongly activated in HTLV-1-infected Tax-negative cells, suggesting a Tax-independent mechanism [130–132]. Moreover, Tax-independent NF-κB activation also happens in Tax-positive cells. Several mechanisms may be involved in Tax-independent NF-κB activation in HTLV-1-infected T cells. It is conceivable that ligation of the T-cell receptor (TCR) following HTLV-1 infection will lead to canonical NF-κB activation. However, if it exists, this is only a minor and transient mechanism, since the TCR and its proximal signaling molecules are quickly down-regulated after antigen ligation [133]. In fact, loss of antigen receptor and its downstream signaling molecules are characteristic and a contributing factor in malignant transformation of lymphocytes mediated by HTLV-1 or directly by the oncogenic NF-κB member c-Rel [134–139]. Possibly, the positive feedback mechanism is the most promising one for Tax-independent NF-κB activation. Largely through NF-κB activation (initially activated by TCR ligation and Tax, and later activated by Tax or Tax-independent mechanisms, see discussion below), HTLV-1 infection induces expression of many NF-κB stimulators and signaling molecules such as TNF, CD40, CD30, and Bcl-3 [140–143]. As discussed previously, TNF is the prototypic stimuli of canonical NF-κB activation, while CD40 and CD30 are potent activators of both canonical and noncanonical NF-κB pathways [144,145]. On the other hand, Bcl-3 binds to p50 or p52 homodimers and transforms them from transcription repressors into activators [27]. Interestingly, CD30 upregulation and its resulting NF-κB activation are hallmarks of anaplastic large cell lymphoma (ALCL) and Hodgkin lymphoma (HL) [145,146]. Other mechanisms involved in Tax-independent NF-κB activation in HTLV-1-infected T cells may be attributed to various stress conditions and epigenetic/genetic alterations caused by HTLV-1 infection. For example, DNA damage, a determining factor in tumorigenesis including ATL leukemogenesis [147,148], can lead to strong NF-κB activation [111]. On the other hand, epigenetic up-regulation of NIK expression and genetic deletions of the p100 C-terminus have recently been detected in certain ATL cells [149–151]. While NIK is a potent activator of both canonical and noncanonical NF-κB pathways [32,55,96], C-terminal deletions of p100 results in constitutive p100 processing and non-canonical NF-κB activation [32,152,153].

### 3.3. Persistent NF-κB Activation by HTLV-1

Unlike the rapid but normally transient activation under physiological conditions, NF-κB activation in HTLV-1-infected cells is aberrantly persistent, whether it is Tax-dependent or -independent or whether it is canonical or noncanonical. A main reason for this abnormal activation is the co-existence and cross-activation of different NF-κB and NF-κB-related signaling pathways. In this way, the tightly controlled activation mechanisms of NF-κB are inappropriately unleashed and the normal termination mechanisms are overridden. Again, the Tax oncoprotein is the primary culprit. First, Tax persistently activates IKK through physical interaction, leading to continuous degradation of IκBα, which controls the early-phase of NF-κB activation, IκBβ and p105, which controls the late-phase of NF-κB activation, as well as constant processing of p100, which controls another late-phase of NF-κB activation (noncanonical pathway) [120,154–158]. Second, Tax binds to and increases the stability and activity of NF-κB and/or prevents NF-κB from binding to its inhibitors [159–167], resulting in a prolonged and elevated activation of NF-κB. Third, Tax directly shuts off the mechanisms that terminate NF-κB activity. For example, Tax prevents nuclear RelA from PDLIM2-mediated ubiquitination and subsequent degradation, although the cost is the sacrifice of Tax itself [168]. Moreover, Tax binds to and recruits NEMO-related protein (NRP/Optineurin) and TAXBP1 to the Golgi-related structures [104]. Although NRP and TAXBP1 are not required for Tax recruit NEMO, the formation of a Tax/NRP/TAXBP1 ternary complex disrupts the A20/TAXBP1 deubiquitinase complex, therefore increasing K63-linked ubiquitination of Tax and possibly also many cellular NF-κB signaling molecules. As discussed previously, K63-linked protein ubiquitination is a key mechanism for signaling complex assembly and NF-κB activation. Fourth, Tax induces expression of NF-κB members, signaling molecules and activators, particularly cytokines, which form a positive feedback loop of NF-κB activation [140–143,159,169–171]. In this way, different NF-κB pathways can be cross-activated. Canonical NF-κB activation induces expression of p100 as well as p100 processing inducers such as CD40 to persistently activate the non-canonical NF-κB pathway [120,140,159]. Non-canonical NF-κB also facilitates canonical NF-κB activation by repressing transcription of the WW domain-containing oxidoreductase (*wwox*) tumor suppressor gene, a specific inhibitor of Tax-induced RelA phosphorylation [172]. In addition to NF-κB, Tax induces many other signaling pathways such as the phosphatidylinositol 3-kinase (PI3K)/AKT and DNA damage signaling pathways, leading a reciprocal enhancement of these pro-oncogenic pathways with NF-κB [8,27,111,173,174]. It should be pointed out that most of these mechanisms also apply to the persistent activation of Tax-independent and -dependent NF-κB.

### 3.4. Differences between Tax-Dependent and Tax-Independent NF-κB Activation by HTLV-1

Both canonical and noncanonical NF-κB signaling pathways are persistently activated in HTLV-1-infected cells regardless of Tax expression. In addition to the common and distinct signaling mechanisms for their activation, Tax-dependent and -independent NF-κB pathways also involve activation of common and distinct NF-κB members. NF-κB members activated in Tax-expressing T cells are predominantly RelA, c-Rel, p50 and p52 [120,159,169], and those in HTLV-1-infected Tax-negative T cells and primary ATL cells are mainly RelA and p50 [131,169]. Consistent with the role of positive feedback mechanisms in persistent NF-κB activation, expression of c-Rel and p100/p52 is induced in Tax-expressing cells while that of p105/p50 mRNA is enhanced in ATL cells [159,169–171]. Activation of common and distinct NF-κB members leads to transcriptional changes, which regulate specific stage of ATL leukemogenesis. For example, c-Rel-mediated activation of IL2 and IL2R may play a critical role in growth, particularly the transition from IL2-dependence to IL2-independence, of HTLV-1-infected T cells during the pre-leukemic stage of ATL [175,176]. On the other hand, p50-dependent induction of activation-induced cytidine deaminase (AID) may contribute to genomic mutations and ATL initiation and development [177].

## 4. NF-κB in ATL Leukemogenesis

### 4.1. Significance of NF-κB in Tax-Mediated Cellular Transformation and ATL Leukemogenesis

The significance of NF-κB activation in ATL leukemogenesis has been suggested since it was linked to HTLV-1 induction of the IL2R in the late 1980s [81,83,84]. The requirement of NF-κB for HTLV-1- or Tax-induced immortalization was largely defined using Tax mutants that are deficient in the activation of either NF-κB or CREB/ATF (cyclic-AMP-response element binding protein/activating transcription factor), a transcription factor responsible for Tax-mediated viral gene expression [178,179]. Surprisingly, these Tax mutant analyses have yielded conflicting results as to whether NF-κB or CREB/ATF activation is critical for Tax-mediated cellular transformation [180–183]. Regardless of the discrepancy, studies using the Tax mutants suggest that NF-κB is important in Tax-induced IL2-dependent or -independent cell growth as well as in HTLV-1-induced T-cell immortalization [184–187]. In addition, inhibition of NF-κB, by silencing NF-κB or its activators IKK and NIK, by over-expressing degradation/processing-resistant forms of IκBα and p100, or by using IKK/NF-κB chemical inhibitors, prevents Tax-mediated cellular transformation and blocks the growth of HTLV-1- or Tax-transformed cells and ATL cells, both in culture and in SCID mice [80,122,132,150,172,180,188–193]. Together, those studies suggest that NF-κB plays a crucial role in HTLV-1/Tax-mediated transformation *in vitro*.

Recently, an *in vivo* role of NF-κB in HTLV-1-mediated tumorigenesis has been demonstrated in two independent studies using two different Tax transgenic mouse models: lymphocyte-restricted Tax transgenic mice and HTLV-1 LTR Tax transgenic mice. The former mice develop a lethal cutaneous disease that shares several features in common with the skin disease that occurs during the preleukemic stage in HTLV-1-infected patients [194], while the latter mice develop different kinds of soft tissue tumors [17,18]. Notably, mice expressing a Tax mutant defective in the activation of NF-κB, but not CREB/ATF, fail to develop the skin disease or any other diseases [194]. More interestingly, genetic knockout of the *nf-kb2* gene alone dramatically delays tumor onset in the HTLV-1 LTR Tax transgenic mice [172]. These *in vivo* studies also suggest that both canonical and non-canonical NF-κB pathways are involved in Tax-induced cellular transformation and tumorigenesis. In this regard, knockdown of either *rela* or *nf-kb2* reduces Tax-induced T-cell proliferation *in vitro* [195]. On the other hand, the transforming activity of Tax2, the homologous Tax protein encoded by HTLV-2, which activates the canonical NF-κB pathway as efficiently as Tax but loses the ability to activate the noncanonical NF-κB pathway, is much lower than that of Tax [196]. Induction of p100 processing by expressing the NIK kinase can restore the transforming activity of Tax2 to a level comparable to that of Tax [196].

### 4.2. Functional Role of NF-κB in Tax-Mediated Cellular Transformation and ATL Leukemogenesis

NF-κB has been suggested to be involved in all stages of ATL leukemogenesis from initiation to invasion and dissemination, through the transcriptional regulation of various tumor-related genes [27]. During the early stages of ATL leukemogenesis, NF-κB induces expression of genes involved in T-cell proliferation and survival such as IL2Rα, IL4, IL6, IL8, IL9, IL13, IL21, IL27, IL15R, CXCR7, MCP-1, CD30, CD40, OX40/OX4OL, miRNA146a, 4-1BB, Bcl-2, Bcl-xL, cIAP, CCD1, CCD2, and CCD6 [81,83,84,140,141,195,197–216]. Activated NF-κB also promotes genetic and epigenetic changes that drive the transformation of HTLV-1-infected T cells via several different mechanisms. The first one involves induction of the ‘mutagenic’ enzyme AID and the epigenetic mediator DNA methyltransferase 1 (DNMT1) [177,217]. The second one depends on transcriptional repression of the cell cycle checkpoint regulator p53 and the DNA repair protein β-polymerase. This function of NF-κB occurs indirectly through RelA-mediated sequestration of the transcriptional coactivators CBP/p300, leading to transcriptional repression of the bHLH transcription factor c-Myb and subsequent inactivation of c-Myb-mediated transcription of p53 and β-polymerase [218–220]. Since the competition for limited CBP/p300 proteins is an important mechanism for the mutual repression of NF-κB and p53 [221–223], CBP/p300 sequestration by RelA may also contribute to the transcriptional inactivation of p53 in HTLV-1-infected cells. Indeed, Tax-induced transcriptional repression of p53 requires IKK-mediated RelA phosphorylation, a modification that is known to promote RelA binding to CBP/p300 [224–226]. Tax also induces a physical interaction between RelA and p53, suggesting another mechanism for NF-κB-mediated p53 inactivation [224]. Consistent with the central role of p53 in tumor suppression and the causative role of NF-κB in tumorigenesis, NF-κB also represses p53 at the protein level using two different mechanisms. First, activated IKK directly phosphorylates p53 to trigger p53 ubiquitination by the β-TrCP ubiquitin ligase and degradation by the proteasome and second, activated NF-κB induces expression of MDM2, a ubiquitin ligase well-known for p53 ubiquitination and degradation [227–229]. Although it remains unknown whether activation of IKK/NF-κB induces degradation of p53 protein in HTLV-1-infected cells, these findings suggest different mechanisms for NF-κB-mediated suppression of p53 for HTLV-1 pathogenesis. Furthermore, NF-κB may contribute to DNA damage and induction of oncogenic mutations indirectly through inflammation-mediated production of reactive oxygen and nitrogen species (ROS and RNS) [27]. Interestingly, NF-κB also activates many other pro-oncogenic molecules/signaling pathways such as c-Myc and PI3K to induce expression of human telomerase reverse transcriptase (hTERT) for the long-term proliferation and clonal expansion of HTLV-1-infected cells that have acquired chromosomal abnormalities [147,174,230,231]. In addition to its role in the initiation and development of ATL, deregulated NF-κB induces expression of many genes involved in tumor progression and metastasis such as matrix metalloproteinase-9 (MMP-9) [232].

## 5. Negative Regulation of Tax

Given the strong oncogenic ability of Tax and its essential role in viral transcription, it is not surprising that this viral oncoprotein is a major target of both humoral and cellular immune responses [233–235]. To evade the host immune surveillance, the virus has evolved several mechanisms that allow Tax to be expressed at the proper time and level. During the late stages of ATL leukemogenesis when Tax functions have been completed or taken over by other mechanisms such as constitutive NF-κB activation, its expression is permanently silenced via genetic mutations or epigenetic repression. Thus, understanding how Tax is regulated will provide important insights into the virus-host interaction, viral latency, ATL leukemogenesis as well as health disparities in HTLV-1 infection. This is particularly important, given that the majority of HTLV-1-infected persons remain lifelong asymptomatic carries and it takes decades for ALT to develop in less than 5% virus carriers.

### 5.1. Repression of Tax by Viral Genes

Besides the *tax* gene, HTLV-1 also encodes several other regulatory/accessory genes including *rex*, *p12*, *p13*, *p30* and *hbz* (Figure 1). Among these gene products, Rex, p30 and HBZ have been reported to negatively regulate the expression and/or activity of Tax. Rex binds to and exports the unspliced and singly spliced viral RNAs, which encode viral structural proteins (env, gag and pol), from the nucleus into the cytoplasm [236,237]. Rex also inhibits splicing of the viral RNAs [238]. In these two ways, Rex increases the expression of viral structural proteins at the expense of Tax and itself, because the Tax and Rex RNAs are generated by a second splicing event from the singly spliced RNA (Figure 1). The p30 protein, on the other hand, inhibits expression of Tax and Rex by trapping the *tax/rex* doubly spliced RNAs in the nucleus [239]. Moreover, p30 blocks Tax-dependent viral gene activation by competing for binding to the transcriptional coactivators CBP/p300 [240]. HBZ (HTLV-1 basic leucine zipper factor), which is encoded by the minus strand of the HTLV-1 proviral genome from 3′-LTR, functions in both RNA and protein forms. The *hbz* RNA promotes T-cell proliferation, and the HBZ protein suppresses Tax-mediated viral transcription by sequestering CREB/ATF, the transcription factor responsible for Tax activation of the HTLV-1 LTR [241–243]. More recent studies suggest that the *hbz* RNA, but not the HBZ protein, increases Tax expression indirectly by down-regulation of p30 RNA [244]. Thus, the *hbz* gene regulates Tax both positively and negatively, depending on its expression form. It should be pointed out that the *hbz* gene induces T-cell lymphoma in mice when it is conditionally expressed in CD4^+^ T cells [245]. Currently, it remains unknown which form (RNA or protein) of the *hbz* gene drives tumorigenesis in the transgenic mice. Whereas the RNA form, but not the protein form, promotes T-cell proliferation *in vitro* [241], the function of HBZ protein in Foxp3 regulation *in vitro* correlates with the increased CD4^+^ Foxp3^+^ Treg cells in mice [245]. Thus, it seems that both forms of the *hbz* gene contribute to tumorigenesis in the transgenic mice. However, *hbz* RNA may be the main functional form in HTLV-1-infected cells, given that *hbz* RNA is strongly expressed in ATL cells and human T cells transduced with HTLV-1 molecular clones [246]. In contrast to the high level of its RNA form, the level of HBZ protein may be very low in infected persons due to high human immune responses toward HBZ [247,248]. The main function of the *hbz* gene in ATL leukemogenesis appears to be maintaining the outgrowth of HTLV-1-transfomed cells [241,243], because it is not required for HTLV-1-mediated T-cell immortalization [249]. Nevertheless, these findings are exciting, as they shed light on the mechanism of how ATL cells maintain the transformed phenotype after Tax is inactivated.

### 5.2. Repression of Tax by Cellular Genes

Apart from the immune responses towards Tax, the mechanism of how Tax is regulated by cellular factors has been rarely studied. One report showed that histone deacetylase 1 (HDAC1) associates with and prevents Tax from binding to the transcriptional coactivator CBP, thereby suppressing Tax activation of viral gene transcription [250]. However, another study suggested that the Tax-HDAC1 interaction benefits viral gene transcription by removing HDAC1 from the viral promoter [251]. Since those studies were performed with over-expressed proteins and in the absence of HTLV-1 infection, the physiological significance of this finding needs to be examined.

More recently, a negative role of PDLIM2 in Tax regulation has clearly been demonstrated. Through a specific Tax-binding motif, PDLIM2 directly shuttles Tax from its activation sites to the nuclear matrix for ubiquitination-mediated degradation when over-expressed and during HTLV-1 infection [79,168]. Consistently, PDLIM2 expression inversely correlates with the stability and activity of Tax in HTLV-1-transformed T cells [168]. Interestingly, PDLIM2 expression is down-regulated in HTLV-I-transformed T cells and in primary ATL cells partially due to methylation of the *pdlim2* promoter [252–254]. Notably, PDLIM2 expression blocks constitutive NF-κB activation, and prevents *in vitro* cell growth and *in vivo* tumorigenesis of Tax-expressing cells and HTLV-1-transformed T cells, whereas PDLIM2 knockout enhances the pathogenic processes [79,168]. These studies suggest that the balance between PDLIM2 and HTLV-1 may determine ATL leukemogenesis. Given its role in terminating NF-κB/RelA activation [78], PDLIM2 may directly target RelA to suppress ATL, particularly during late stages of leukemogenesis when Tax expression is lost. In support of this, PDLIM2 expression is epigenetically repressed in several tumors such as breast and colon cancers, and expression of exogenous PDLIM2 or re-induction of endogenous PDLIM2 inhibits constitutive NF-κB activation and suppresses *in vitro* anchorage-independent growth and *in vivo* tumor formation of those malignant cells [253,254].

## 6. Conclusions and Perspectives

Over the past three decades, significant progress has been made toward understanding the molecular mechanism of constitutive NF-κB activation and its functional role in Tax-mediated tumorigenesis and ATL leukemogenesis. These studies have greatly enhanced our knowledge of NF-κB signaling regulation and NF-κB-associated tumorigenesis beyond ATL. However, many key issues have not yet been addressed. First, it is largely unknown how IKK is activated by the Tax-IKK interaction and whether Tax-independent IKK/NF-κB activation in HTLV-1-infected T cells is reminiscent of cellular mechanisms such as those induced by cytokines, oxidative stress and genetic stress. Second, there is still no convincing evidence for a functional role of NF-κB pathways, particularly different NF-κB family members, in Tax-mediated tumorigenesis or ATL leukemogenesis. Most functional studies have focused on the *in vitro* effects on Tax-induced cell growth and immortalization using IKK or NF-κB inhibitors (most of them not completely NF-κB specific, and IKK has many functions independent of NF-κB activation) or Tax mutants defective in NF-κB but not CREB/ATF activation. However, Tax has many functions beyond NF-κB and CREB/ATF. Moreover, the functions of Tax are highly sensitive to structural changes [178,179]. The loss-of-function studies through Tax mutations may be artificial. Third, it remains largely unknown how NF-κB cooperates with other signaling pathways in tumorigenesis. In this regard, NF-κB is known to crosstalk with many other tumor-related signaling pathways such as autophagy and PI3K signaling pathways [255–257]. Fourth, most studies focus on the net effect of NF-κB activation on cell growth and tumor tumorigenesis. As an old Chinese saying goes, everything has yin (negative) and yang (positive), two opposite aspects, and so does NF-κB. Although NF-κB activation contributes to tumorigenesis in general, it may also play a negative role at certain stages of tumorigenesis and even exert a net negative effect on tumorigenesis in certain situations. One mechanism of NF-κB-mediated tumor suppression involves its original function in immunity and immunosurveillance [27]. Moreover, Tax-activated NF-κB may also lead to cell apoptosis [258]. Currently, it is largely unknown how the anti-tumor activity of NF-κB is suppressed and converted to be pro-tumorigenic for ATL development. It is possible that various cytokines/chemokines and other factors involved in immune responses also stimulate growth and migration of pre-tumor and tumor cells, in addition to immune cells [27]. In this regard, HTLV-1-infected T cells are in a unique position, because they are part of the immune system. It is also possible that human immune activation may induce Tax expression and reactivate latent HTLV-1, thereby leading to ATL development or other viral pathogenesis [259]. Fifth, very few downstream targets of NF-κB that play a critical role in tumorigenesis have been clearly and comprehensively identified. Sixth, possibly the most important and interesting question in the HTLV-1 field is how the Tax oncoprotein and the *hbz* gene cooperate and contribute to the pathogenesis of ATL and other HTLV-1-associated diseases. Finally, there is a lack of a systematic analysis of the correlations between ATL development and viral gene expression, PDLIM2 repression and NF-κB activation. Future genetic studies, particularly those using inducible and conditional transgenic mice, and computational modeling analysis will help to understand the complex and dynamic role of NF-κB in ATL leukemogenesis and other human tumors, and help to design personalized treatments for cancer patients.
